# Supplementary material for: Identification of RNA-Binding Proteins as Targetable Putative Oncogenes in Neuroblastoma
Source: Int J Mol Sci. 2020 Jul 19;21(14):5098. doi: 10.3390/ijms21145098 (PMC7403987; doi:10.3390/ijms21145098)
Supplement: Supplementary file 1 [file ijms-21-05098-s001.zip › ijms-797756-S.pdf]

International Journal of
Molecular Sciences

MDPI

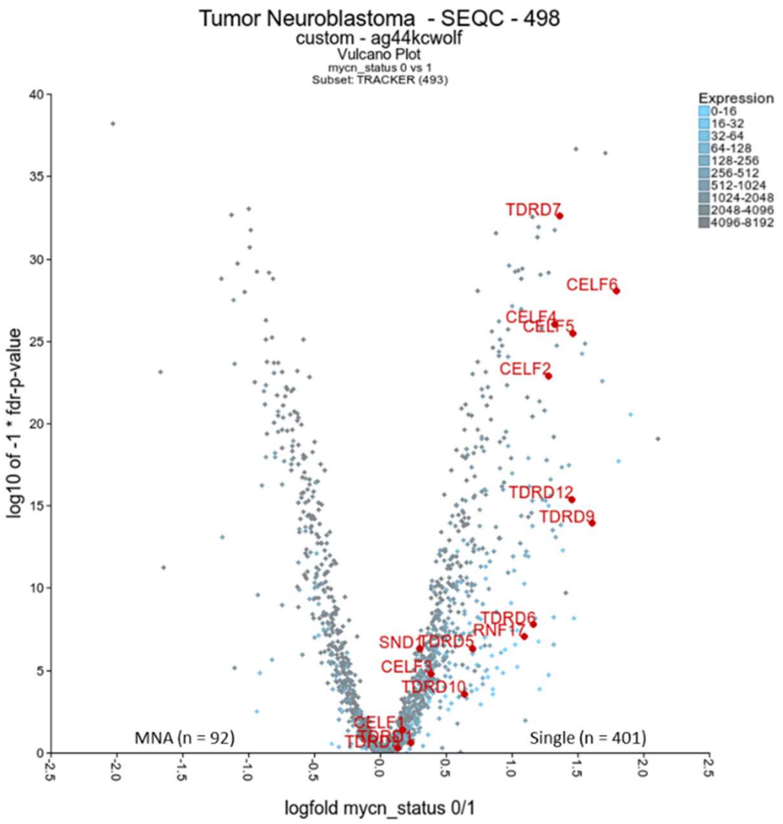

Figure S1. Differential expression of RBPs in clinically relevant neuroblastoma groupings (annotations focus on tumour-suppressive RBP families). This is a similar figure to 1A with different annotations used. CELF, TDRD and CPEB gene families have been annotated where possible. MNA = *MYCN* amplified, single = non-amplified *MYCN* status. RNF17 is a TDRD family member.

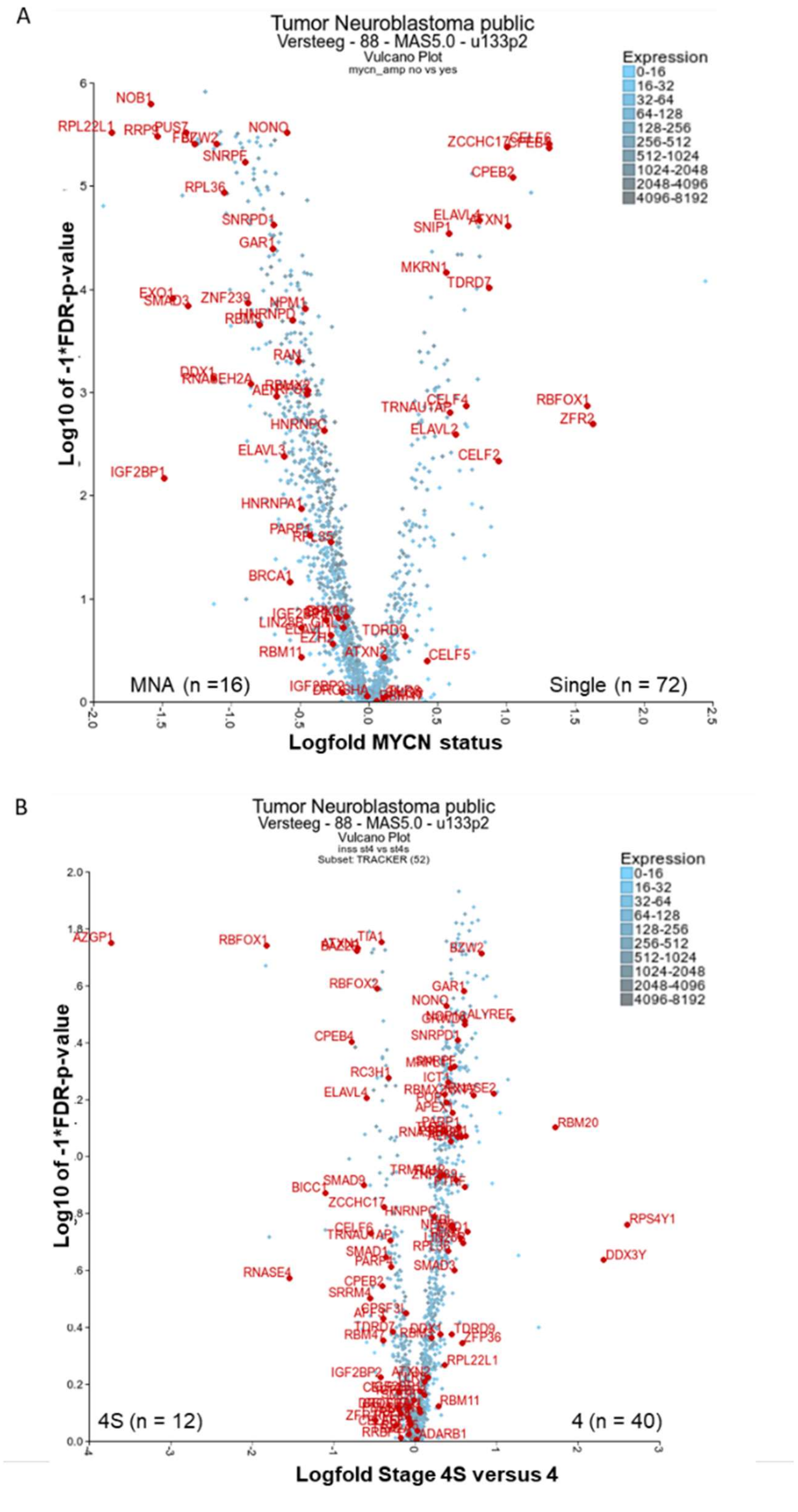

Figure S2. Differential expression of RBPs in clinically relevant neuroblastoma groupings (second cohort). The RBP gene list from Gerstberger et al. 2014 [16] was used to filter for RBP mRNAs and then compare (**A**) *MYCN* status of tumours or (**B**) Stages 4 and 4S. Both the analyses were displayed as a volcano plot using the two groups plotter option of the R2 the visualisation tool (https://r2.amc.nl). Versteeg cohort with the custom normalisation option was used on the R2 platform (GEO accession: GSE16476). mRNAs that were annotated in Figure 1 have been annotated where possible. mRNAs had to have a minimum of 20 expression units and be present in 20 tumours.

Statistical analysis was conducted with the Kruskal rank-based test, and FDR correction of p-values was performed. MNA = *MYCN* amplified, single = non-amplified *MYCN* status.

© 2020 by the authors. Submitted for possible open access publication under the terms and conditions of the Creative Commons Attribution (CC BY) license (http://creativecommons.org/licenses/by/4.0/).
